# Editorial: A comprehensive look at biomarkers in neurodegenerative diseases: from early diagnosis to treatment response assessment

**DOI:** 10.3389/fnagi.2025.1642793

**Published:** 2025-06-23

**Authors:** Riccardo Pascuzzo, Fulvia Palesi, Yi-Min Wan, Federico Angelo Cazzaniga

**Affiliations:** ^1^Neuroradiology Unit, Fondazione IRCCS Istituto Neurologico Carlo Besta, Milan, Italy; ^2^Department of Brain and Behavioral Sciences, University of Pavia, Pavia, Italy; ^3^Department of Psychiatry, Ng Teng Fong General Hospital, Singapore, Singapore; ^4^Unit of Laboratory Medicine–Laboratory of Clinical Pathology, Fondazione IRCCS Istituto Neurologico Carlo Besta, Milan, Italy

**Keywords:** Alzheimer's disease, Parkinson's disease, cognitive impairment, dementia, diagnosis, risk factor, molecular mechanism

## 1 Introduction

Neurodegenerative diseases (NDs), including Alzheimer's disease (AD) and Parkinson's disease (PD), pose an escalating global health crisis, affecting millions worldwide and placing an immense burden on healthcare systems (GBD 2021 Nervous System Disorders Collaborators, 2024). A shared hallmark of many NDs is the misfolding, aggregation, and accumulation of specific proteins in the brain, events that often precede the onset of clinical symptoms (Sweeney et al., 2017). The identification and validation of reliable biomarkers that can detect these underlying pathological processes are crucial for improving diagnosis, predicting disease progression, and monitoring the efficacy of therapeutic interventions.

This Research Topic brings together a collection of original research articles and reviews that significantly contribute to our understanding of the aging brain and the mechanisms underlying NDs. The studies encompass a range of methodologies, from molecular investigations to clinical assessments and computational analyses, offering a multifaceted perspective of the role of different biomarkers across a wide spectrum of NDs, including tauopathies, synucleinopathies, prion diseases, and other proteinopathies associated with dementia and cognitive impairment.

## 2 Diagnostic biomarkers of AD and cognitive impairment

Early and accurate diagnosis remains a critical goal in managing neurodegenerative conditions, particularly AD, where pathological changes such as amyloid-β accumulation and tau pathology begin many years before the onset of clinical symptoms. Detecting these molecular alterations at the preclinical or prodromal stage is essential not only for enabling timely intervention and care planning but also for the effective recruitment and stratification of participants in clinical trials of disease-modifying therapies (Hansson, 2021). Nonetheless, biomarker positivity alone, particularly isolated amyloid or tau abnormalities, may not deterministically predict clinical symptom onset or fully capture the complex and multifactorial nature of AD pathology. This underscores the necessity of continued research into other contributing mechanisms, as well as longitudinal studies to more accurately assess lifetime risk and optimize preventive strategies (Dubois et al., 2024; Villain and Planche, 2024).

In this Research Topic, Zhang et al. explored the potential of novel ocular biomarkers for the early identification of mild cognitive impairment (MCI), highlighting the promise of non-invasive methods for both patients and healthcare providers. Similarly, Ibrahim et al. delved into the relationship between retinal microvascular density and inner thickness in AD and MCI, seeking to unravel significant retina map parameters associated with cognitive decline. Complementing these efforts, Ge et al. presented an EEG-based framework for the automated discrimination of conversion to AD in patients with amnestic MCI, providing robust longitudinal evidence for early prediction. Moreover, Wang et al. further contributed to AD diagnostics by investigating rhythmic power changes and phase differences using low-density EEG. Additionally, Xu et al. explored the diagnostic potential of urinary CX3CL1 for amnestic MCI and AD, suggesting a novel non-invasive candidate biomarker. In a study of Hsieh et al., the authors conducted a longitudinal assessment of plasma biomarkers for the early detection of cognitive changes in subjective cognitive decline (SCD), finding higher plasma Aβ42 levels in individuals with SCD compared to healthy controls.

Neuroimaging techniques provide another powerful avenue for biomarker discovery, offering structural, functional, and molecular insights into NDs (Young et al., 2020). In this Research Topic, Park et al. presented a deep learning-based quantification of brain atrophy using 2D T1-weighted MRI for AD classification, demonstrating the potential of artificial intelligence in enhancing the diagnostic ability of neuroimaging. In another study, Ruan et al. used multichannel functional near-infrared spectroscopy (fNIRS) to study cortical activation in elderly patients with AD dementia during working memory tasks, providing valuable data on functional brain changes.

## 3 Diagnostic biomarkers of PD

Beyond AD, accurate and early clinical diagnosis of PD will be increasingly crucial especially for future disease-modifying clinical trials (Koga et al., 2021) and the development of more personalized treatments. In this Research Topic, Chen et al. investigated the associations of motor and neuropsychiatric symptoms with comorbidities in prodromal PD, shedding light on the complex early manifestations of this disorder. Additionally, Li et al. focused on the reduced maximal range of ocular movements in PD and its response to acute levodopa challenge, identifying a potential clinical marker. Furthermore, Gu et al. demonstrated that serum neurofilament light chain (NfL) levels are significantly higher in PD patients and can predict cognitive impairment within a 2-year timeframe, suggesting NfL as a feasible biomarker. Finally, Lu et al. examined the correlations of erythrocytic oligomer α-synuclein levels with age, sex, and clinical variables in PD patients, exploring a potential peripheral biomarker.

## 4 Biomarkers and molecular mechanisms of NDs

While the identification of diagnostic biomarkers is essential, a deeper understanding of the molecular and cellular mechanisms driving neurodegenerative diseases is equally critical for informing therapeutic development (Wareham et al., 2022). Dissecting the complex interplay between genetic regulation, immune dysregulation, and neuronal dysfunction can uncover novel targets and pathways for intervention. Several contributions in this Research Topic offer important advances in elucidating these underlying mechanisms: Lin et al. utilized single-cell RNA sequencing to identify altered immune cell types and molecular mechanisms in AD progression, providing insights into the role of the immune system; Filomena et al. employed an integrated gene co-expression network analysis of hippocampus and fusiform gyrus RNA-seq datasets to identify deregulated long non-coding RNAs in AD, potentially revealing new therapeutic targets; Amelimojarad et al. reviewed the emerging role of brain neuroinflammatory responses in AD patients, highlighting chronic inflammation's contribution to AD pathology. In a model organism approach, Xue et al. studied olfactory dysfunction as an early pathogenic indicator in *C. elegans* models of AD and polyglutamine diseases, providing insights into early disease mechanisms. Furthermore, Liao et al. investigated the expression of Toll-like receptors in the cerebellum during the pathogenesis of prion disease in mice, contributing to our understanding of immune responses in neurodegeneration. Finally, Liang et al. examined whether serum TRPA1 mediates the association between olfactory function and cognitive function, suggesting a link between sensory and cognitive decline.

## 5 Risk factors and treatment response assessment in NDs

This Research Topic also addresses broader risk factors, the identification of which is crucial for effective prevention and targeted risk mitigation (Livingston et al., 2024), alongside strategies aimed at modifying disease progression and managing clinical symptoms. In a study of Sheng et al., the authors investigated the relationship between hyperthyroidism, hypothyroidism, thyroid-stimulating hormone, and dementia risk, utilizing both observational data and Mendelian randomization analysis to explore the role of thyroid function. Furthermore, Cheng et al. explored the connection between liver fibrosis and PD, highlighting potential systemic influences on neurodegenerative processes.

Among the studies assessing treatment response, Zhao et al. used fNIRS to investigate the effects of repetitive transcranial magnetic stimulation (rTMS) in AD patients with depression, observing that rTMS led to a reduced prefrontal activation during a verbal fluency task and that this reduction was associated with an improvement in depressive symptoms. Finally, Manap et al. provided a comprehensive review on the current trends of effective diagnosis and therapeutics for AD, offering an overview of the latest advancements.

## 6 Conclusions

This Research Topic provides a comprehensive overview of the current landscape of biomarker research in NDs. The contributing articles highlight the diverse approaches being employed to identify and validate biomarkers for early diagnosis, differential diagnosis, and understanding disease progression across various NDs, including AD, PD, and prion diseases. The findings presented, ranging from the investigation of novel fluid and genetic markers to the application of imaging and artificial intelligence, as well as the exploration of systemic and immunological factors, collectively advance our understanding of the complex molecular mechanisms underlying these debilitating conditions. Continued research in this critical area holds immense promise for the development of more effective diagnostic tools and ultimately, disease-modifying therapies that can improve the lives of individuals affected by NDs.
